# Imaging the surface potential at the steps on the rutile TiO_2_(110) surface by Kelvin probe force microscopy

**DOI:** 10.3762/bjnano.10.122

**Published:** 2019-06-13

**Authors:** Masato Miyazaki, Huan Fei Wen, Quanzhen Zhang, Yuuki Adachi, Jan Brndiar, Ivan Štich, Yan Jun Li, Yasuhiro Sugawara

**Affiliations:** 1 Department of Applied Physics, Graduate School of Engineering, Osaka University, 2-1 Yamdaoka, Suita, Osaka 565-0871, Japan; 2 Institute of Physics, CCMS, Slovak Academy of Sciences, Bratislava, Slovakia

**Keywords:** catalyst, Kelvin probe force microscopy, Smoluchowski effect, step, titanium dioxide

## Abstract

Although step structures have generally been considered to be active sites, their role on a TiO_2_ surface in catalytic reactions is poorly understood. In this study, we measured the contact potential difference around the steps on a rutile TiO_2_(110)-(1 × 1) surface with O_2_ exposure using Kelvin probe force microscopy. A drop in contact potential difference was observed at the steps, indicating that the work function locally decreased. Moreover, for the first time, we found that the drop in contact potential difference at a <1−11> step was larger than that at a <001> step. We propose a model for interpreting the surface potential at the steps by combining the upward dipole moment, in analogy to the Smoluchowski effect, and the local dipole moment of surface atoms. This local change in surface potential provides insight into the important role of the steps in the catalytic reaction.

## Introduction

Titanium dioxide (TiO_2_) has attracted considerable interest for its promising applications as a photocatalyst and as catalyst support, as well as in gas sensors [1–7]. The catalytic activity can be enhanced by the presence of defects, such as oxygen vacancies (O_v_), Ti interstitials (Ti_int_) [8], and crystal steps. TiO_2_ is an n-type semiconductor because of these defects. In addition, reactive oxygen species, such as OH and H_2_O_2_ (compounds with an oxygen adatom (O_ad_) and hydrogen (H) atoms on the surface), play an important role in catalytic reactions, and many studies about the adsorption state and the reaction processes have been performed [9–12]. In general, crystal steps exhibit a high reactivity [13–15] because of their low coordination and unique charge distribution [16–19]. In the case of TiO_2_, steps act as preferential sites for the adsorption of molecules and metal clusters [20–22], as active sites for catalytic reactions [23–25], and as the central elements of surface reconstructions [26–27]. Concerning the charge properties of steps on TiO_2_, it has been measured with using ultraviolet photoelectron spectroscopy (UPS) that surfaces with a high step density have a lower work function than surfaces with a low step density [28]. The local change in the surface potential at steps on TiO_2_ has been observed with a lateral resolution of several nanometers by Kelvin probe force microscopy (KPFM) [29–30]. However, the dependence of surface potential on direction and structure of steps such as [001], 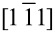 and 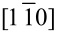 has not yet been clarified.

**Figure 1:**
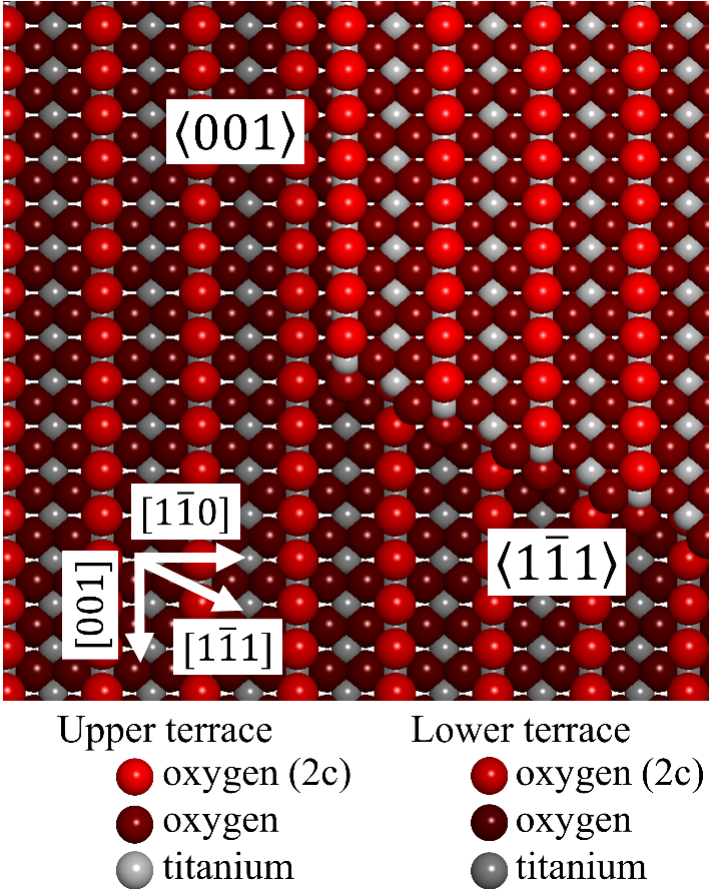
Ball model of TiO_2_(110)-(1 × 1) surface with two step structures of 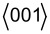 and 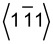.

In scanning tunneling microscopy (STM) [31] studies, three typical steps running along the [001], 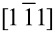, and 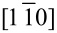 directions were observed [32–35], as shown in Figure 1. Density functional theory (DFT) calculations have provided the step configurations and their relative stabilities [36–38]. The 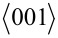 steps have two types of structures: steps terminated with bridging oxygen atoms 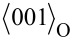 and titanium-terminated steps 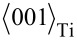 with in-plane oxygen atoms exposed at the steps. The 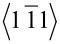 steps are considered to have two types of structures: bulk-terminated steps 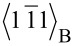 and reconstructed steps 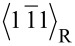 with one additional TiO_2_ unit. The 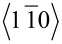 steps are metastable and rarely observed in common sample preparation. The 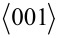 steps showed a higher photodegradation activity than the 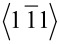 steps for aqueous solutions of methylene blue [23], indicating that the different step structures have different catalytic activities.

KPFM measures the contact potential difference (CPD), corresponding to the difference in work function between the tip and the sample, on the basis of atomic force microscopy (AFM) [39–40]. Since the CPD strongly depends on the charge distribution on the surface, KPFM allows us to investigate the electrostatic properties of surfaces [41–43].

In this study, we measured the CPD around the steps on rutile TiO_2_(110) surfaces with O_2_ exposure using KPFM and observed the drop in CPD at the steps, indicating that the work function locally decreased. Moreover, we found, for the first time, that the drop in CPD at a 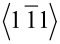 step was larger than that at a 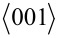 step. We discuss a possible origin of the change in CPD and propose a simple model for interpreting the local surface potential at the steps with the help of surface charge redistribution, in analogy to the Smoluchowski effect, and the local dipole moment of surface atoms supported by the DFT simulation.

## Experimental

The experiments were carried out with a custom-built ultrahigh-vacuum noncontact atomic force microscopy (NC-AFM) system operated at a temperature of 78 K with a base pressure below 4 × 10^−11^ mbar. The NC-AFM system was operated in the frequency-modulation mode [44] with a constant cantilever oscillation amplitude (5 Å). The cantilever deflection was measured using an optical beam deflection method [45]. The images were obtained using a commercial Ir-coated Si cantilever (NANOSENSORS) with a resonant frequency of 804 kHz and 808 kHz and a spring constant of 1500 N/m. Before the experiments, the tip was cleaned by Ar^+^ sputtering (1 keV, 6.7 × 10^−7^ mbar, 5 min) and annealing (600 K, less than 2.7 × 10^−10^ mbar, 20 min) to remove the native oxide layer and other contaminants.

A clean rutile TiO_2_(110) crystal (provided by Furuuchi Chemical Corporation) was prepared by dozens of cycles of Ar^+^ sputtering (1 keV, 1.3 × 10^−6^ mbar, 10 min) and annealing (993 K, less than 2.7 × 10^−10^ mbar, 30 min). After the surface preparation, the color of the surface became the dark-blue, which implies that TiO_2_ is in a highly reduced state [2]. After cooling to room temperature, the sample was exposed to O_2_ (300 K, less than 2.7 × 10^−9^ mbar, 2 min) because O_2_ affects the oxidation state and thus the electron density on the surface.

KPFM measurements were carried out in the frequency-modulation mode [46]. An ac bias voltage (*V*_AC_) at the frequency *f*_AC_ and a dc bias voltage (*V*_DC_) were applied to the sample. *V*_DC_ was adjusted to compensate the *f*_AC_ component of the electrostatic force, providing the CPD value (*V*_CPD_). The topography and CPD were measured sequentially using the lift-mode technique to minimize crosstalk [47]. In this scanning mode, the topography (*z*) is scanned in the first trace using AFM and immediately retraced with a given offset, *z* + offset, using KPFM to measure the CPD. The given offset (100 pm) was applied to avoid the influence of a phantom force [48–49] or induced dipole moments [50].

Force spectroscopy measurements [51] were performed by recording the frequency shift (Δ*f*) as a function of the tip–sample distance. The long-range contribution (Δ*f*_LR_), van der Waals or electrostatic forces, to the Δ*f* curve was fitted to the inverse power law *z*^−^*^n^* [52]. By subtracting the Δ*f*_LR_ curve from the Δ*f* curve, we obtained the short-range contribution (Δ*f*_SR_). Finally, the Δ*f*_SR_ curve was converted to the short-range force (*F*_SR_) using the Sader inversion algorithm [53].

We obtained *Z*–*X* KPFM data perpendicular to the surface measured. The tip was made to approach the surface with an assigned Δ*f* set point and sample bias *V*_DC_ = 0 V to determine the reference height. Then, the CPD was measured 2 nm above the reference height. This measurement was performed along a grid line of 200 points. The atom-tracking technique [54] was employed to reduce the effects of thermal drift.

## Results and Discussion

### CPD measurements around the steps

**Figure 2:**
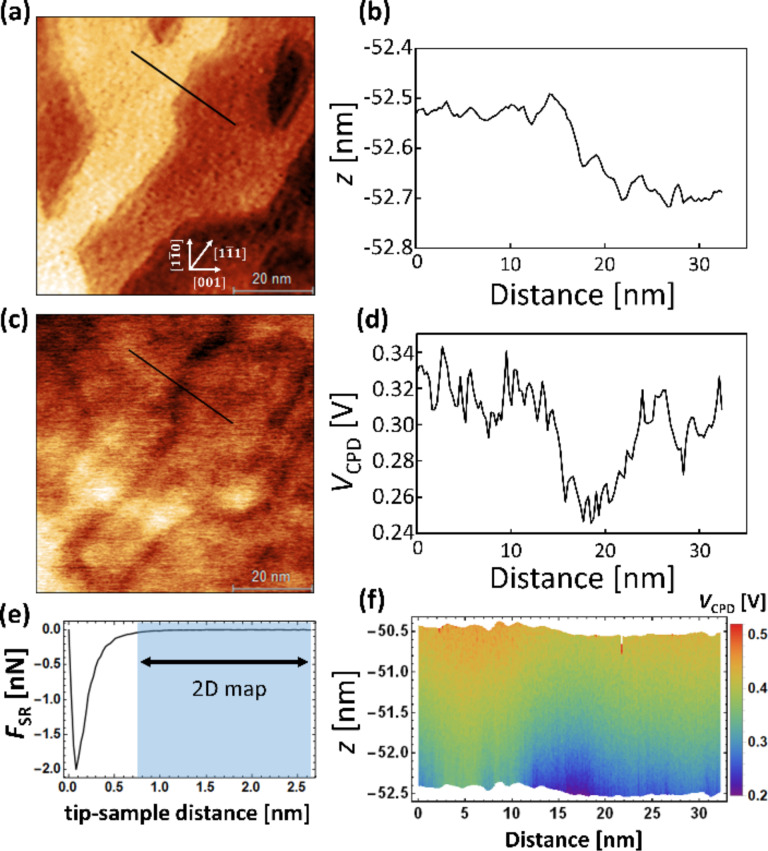
AFM/KPFM images (70 × 70 nm^2^) of the TiO_2_(110) surface after O_2_ exposure. (a) Topographic image and (b) height profile. (c) CPD image and (d) CPD line profile. Line profiles were taken along the black line in (a) and (c). (e) Short-range force curve measured on the terrace. (f) *Z*–*X* KPFM data obtained along the black line in (a). The measured *z*-region corresponds to the blue square in (e). The acquisition parameters: *Q* = 21134, Δ*f* = −150 Hz, *V*_DC_ = 0 V, *f*_AC_ = 180 Hz and *V*_AC_ = 1 V.

A topographic image obtained in the lift-mode and the height profile are shown, respectively, in Figure 2a and Figure 2b. Flat terraces and the steps predominantly parallel to the 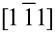 direction, 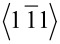, were observed. The short steps parallel to the [001] direction, 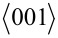, were also observed. The result that there are more 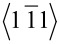 steps than 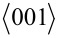 steps is in good agreement with previous results [36–38]. From the height profile perpendicular to the 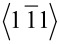 direction (black line in Figure 2a), the height of the step was about 200 pm. The measured step height of 200 pm was smaller than real step height of 325 pm [32], which can be explained by the large tip–sample distance. Actually, at the large tip–sample distances, the van der Waals force is dominant and the contribution of the force from the tip apex becomes weak and the force from the rest of the cantilever becomes significant. Therefore, the observed height of step was smaller than that of the real one. Before the experiments, we verified that the distance calibration of the probe was correct. In the experiment, even though the scan speed changed, the height of the step edges remained constant, indicating that the height of the step was not affected by a potential non-linear response of the *z*-piezo actuator. Thus, the observed steps of 200 pm height must be monatomic steps. The CPD image obtained in the lift-mode and the CPD profile are shown, respectively, in Figure 2c and Figure 2d. The CPD image shows that the CPD decreases at the steps, indicating that the work function locally decreases at the steps. The CPD profile shows that the drop in CPD at the steps is about 70 mV, which is consistent with a previous study, in which surfaces with a high step density were found to have a lower work function than surfaces with a low step density [28]. The drop in CPD at the steps is not due to a feedback error since the forward and backward curves of the topography and CPD profiles have the same configuration. Moreover, we performed the same CPD measurements in the lift-mode on the TiO_2_(110) surface without O_2_ exposure, and it was similarly observed that the work function decreased at the steps.

Here, we discuss the possible origin of the change in CPD, for which several factors can be considered: a phantom force derived from the flow of a tunneling current [48–49], the local adsorption of molecules, the localization of defect states [55–56], the induced dipole moment [50], the unintentional change in tip–sample distance [57], the electron redistribution due to orbital splitting [18] and the Smoluchowski effect [58].

First, we analyze the influence of a tunneling current flowing between the tip and the sample, i.e., a phantom force. The probability of tunneling depends on the atomic site. This may influence the observed CPD value, but it should be excluded as the origin of the change in CPD because no tunneling current was measured during CPD measurements.

Second, we consider the effect of the local adsorption of molecules. Since the sample was exposed to O_2_, O_2_ might adsorb on the steps and change the CPD. However, this effect should be excluded because adsorbed negatively charged O_2_ should increase the CPD, but we observed a decrease in CPD at the steps. Moreover, since no previous studies have shown that O_2_ preferentially adsorbs at the steps on a rutile TiO_2_(110) surface, we consider that the effect of the localized adsorption of O_2_ is negligible. In addition, we also observed a drop in CPD on the surface without O_2_ exposure, indicating that the drop in CPD is not due to O_2_ adsorption.

Third, we analyze the influence of the local pinning of the Fermi level due to defect states [55–56]. In the case of n-type semiconductors, negatively charged defect states derived from donor atoms may localize at the steps and increase the work function due to upward band bending. In contrast, for p-type semiconductors positively charged defect states from acceptor atoms may localize at the steps and decrease the work function due to downward band bending. However, we can rule out this effect because the work function decreased at the steps although TiO_2_ is an n-type semiconductor. It can be inferred that the local surface potential at the steps does not change simply because the sample is an n- or p-type material.

Fourth, we consider the effect of the induced dipole moment due to the chemical bond between the tip apex and surface atoms [50]. The induced dipole moment appears only in the short-range regime. As shown in Figure 2e and Figure 2f, we measured the *F*_SR_ curve on the terrace and then obtained the *Z*–*X* KPFM data along the black line in Figure 2a in the long-range regime (blue region in Figure 2e), where the *F*_SR_ was almost zero. We can see the step configuration at a lateral distance of 15 nm and a dark contrast at the steps, which indicates that the CPD decreases at the steps, i.e., the work function decreases locally. As a result, although the induced dipole moment may influence the observed CPD value, this influence should be excluded because a drop in work function was observed in the long-range regime, where chemical bonds are not formed.

Fifth, we analyze the influence of an unintentional change in tip–sample distance due to the long-range force interacting with a larger effective area or volume when the tip is on the lower terrace near the steps [57]. This may change the measured CPD value since the CPD depends on the tip–sample distance. The *Z*–*X* KPFM data (Figure 2f) shows that the CPD increased with increasing in tip–sample distance, which is the opposite behavior to the decrease in CPD at the steps (Figure 2c and Figure 2e). Therefore, we can rule out the influence of an unintentional increase of the tip–sample distance. In addition, the influence of the electrostatic force on the topography measurements has been investigated. As a result, the topography at the step edges at *V*_DC_ = 0 V was not corrupted by the influence of the electrostatic forces because of the CPD difference on terrace and step. From Figure 2f, we found that the CPD on the terrace has a similar dependence on the tip–sample distance as on the steps when measured on the scale of nanometers.

The topography shows blurred steps in Figure 2a. This does not originate from a double tip. In general, the incorrect step height in topography might affect the measured CPD value at the steps. However, in our experiment, the drop in CPD at the steps in Figure 2f was distributed over a distance greater than 1 nm above the surface (*z*-direction). Therefore, the drop in CPD at the step reflected the intrinsic surface potential.

There might occur an electron distribution by orbit splitting, because the coordination number is smaller on the step edges than on the terrace [18]. Hence, we discuss a possible effect of orbit splitting. In STM studies, the local density of states (LDOS) of the orbit splitting has a large influence on the dipole moment of atomic species at the step edges. Although the orbit splitting has a big influence on the tunneling current, the influence on the measurement of the surface potential in KPFM is expected to be smaller than that of the electrostatic potential of atomic species at the step edges. To clarify the influence of the orbit splitting for CPD, insights from DFT calculations are necessary.

### Comparison between <001> and <1−11> steps

**Figure 3:**
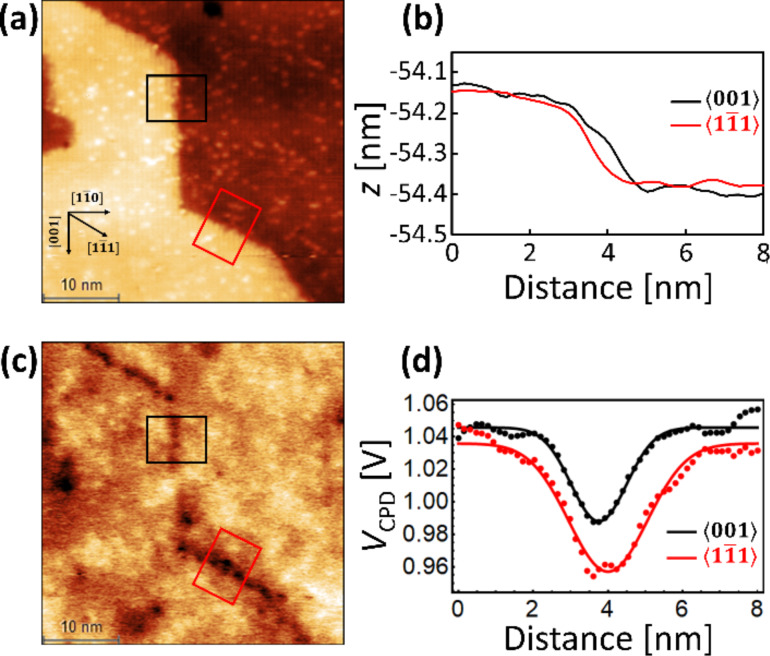
AFM/KPFM images (40 × 40 nm^2^) of TiO_2_ after O_2_ exposure, showing 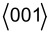 and 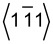 steps. (a) Topographic image and (b) average height profiles. (c) CPD image and (d) average CPD profiles. These line profiles show average values from 40 lines perpendicular to the steps in the black and red rectangles in (a) and (c). Dots are experimental results in (d) and solid lines are Gaussian fits of the experimental data. The acquisition parameters: Q = 29435, Δ*f* = −100 Hz, *V*_DC_ = 0 V, *f*_AC_ = 170 Hz and *V*_AC_ = 1 V.

For further understanding of the electronic properties of the steps, we performed CPD measurements in an area with a sufficiently long 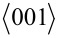 step. The topographic image obtained in the lift-mode and average height profiles are shown, respectively, in Figure 3a and Figure 3b. The topographic image shows that both 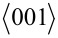 and 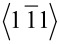 steps are formed. In this image, the bright spots on the surface are adsorbed oxygen species or other adsorbates. The profiles of the average height show that the height of both 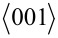 and 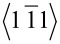 steps was about 260 pm, which is smaller than the known height of 325 pm because of the large tip–sample distance. As shown by the CPD image in Figure 3c, the CPD decreases at both 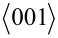 and 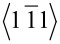 steps, indicating that the work function locally decreases. The average CPD profiles (Figure 3d) show that the CPD values at the 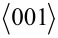 and 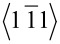 steps dropped to 58 and 79 mV, respectively. These values are the CPD differences on the terrace (Gaussian fit) and the step (minimum of the Gaussian fit). The drop in CPD value on the 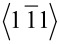 step (79 mV) in Figure 3d is slightly different from the value (70 mV) in Figure 2d because a different tip was used in the experiment. In Figure 3d, the difference in the CPD drop between the 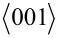 and 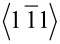 steps is 21 mV, which is larger than the difference based on the Gaussian fit of the 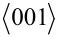 and 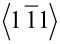 steps (9.8 mV). The KPFM measurements were performed at a sufficiently slow speed to minimize the influence of the differences in angle between the directions of the step and the fast scan.

**Figure 4:**
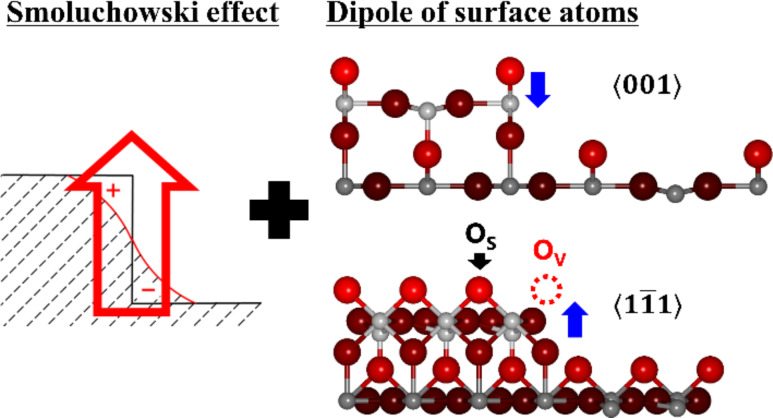
Schematic model for interpreting the local surface potential at the steps that combines the upward dipole moment, in analogy to the Smoluchowski effect, and the local atomic dipole moment. Blue arrows indicate the direction of the local atomic dipole moment.

Now we discuss the influence of charge redistribution due to the presence of different steps in analogy to the Smoluchowski effect [58]. We propose a simple model for interpreting the local surface potential at the steps as a result of charge redistribution and the local dipole moment of surface atoms (Figure 4). The Smoluchowski effect is the theory of the charge distribution at sharp contours such as steps. An upward dipole moment is created at the steps due to the incomplete screening of positive ion cores by conduction electrons because the electronic density cannot follow the step configuration, locally reducing the work function at the steps. Since the Smoluchowski effect depends on the step configuration, the degree of the upward dipole moment should be different at the 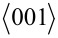 and the 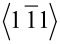 steps.

Generally, the work function depends on the crystal faces [59], and the (110) and the (100) surfaces have a different work function for rutile TiO_2_ [60], which was explained by the difference in the electric double layer formed on the surface. Since the origin of the double layer and the Smoluchowski effect is essentially the same, we think that the Smoluchowski effect may be suitable for explaining the change in surface potential at the steps (Smoluchowski-like dipole). In our experiment, free carriers existed on the surface because a tunneling current flowed on the surface when the bias was applied, indicating that TiO_2_ surface was not insulated by O_2_ exposure. Furthermore, the electrostatic potential distribution at the steps of well-ordered Si(111) semiconductor surfaces has been explained by the Smoluchowski effect [19]. Therefore, the change in CPD at steps of n-type TiO_2_ might be explained by the Smoluchowski effect. The Smoluchowski effect is well known for metals that have an orders of magnitude higher density of free electrons than semiconductors. In this study there is not enough experimental evidence to conclude that the Smoluchowski effect is responsible for the observed effect; further experiments or theoretical investigations such as DFT calculations would be required.

In addition to the Smoluchowski-like dipole, the local dipole moments of surface atoms also exist on the surface, creating a local atomic dipole. On one hand, for both 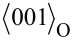 and 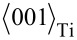, since the negatively charged oxygen atom is exposed at the steps [36–38], the local atomic dipole that is formed points downward in the opposite direction to the Smoluchowski-like dipole (Figure 4). On the other hand, for both 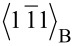 and 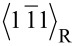, since O_v_ sites exist at the step edges and the positively charged titanium atom is exposed at the steps, the local atomic dipole that is formed points upward in the same direction as the Smoluchowski-like dipole. Moreover, the oxygen atom labeled as O_s_ in Figure 4 might be an oxygen vacancy (O_sv_) [24–25,35], causing another Ti atom to be exposed enhancing the upward local atomic dipole. According to Sasahara et al. [29], the dipole moments are formed in the central direction (horizontal direction) of the upper step by relaxation into the bulk direction of the Ti atom on the step edge. However, the dipole created by the surface atom is not formed in the central direction of the upper step edge.

As a result, the local surface potential at the steps should be interpreted as a combination of the Smoluchowski-like dipole and the local atomic dipole. These effects explain why the drop in CPD at the 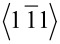 step is larger than that at the 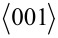 step. In addition, since the CPD decreased on both 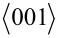 and 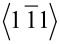 steps, the Smoluchowski-like dipole has a larger effect than the local atomic dipole. It is pointed out that the dipole of 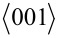 and 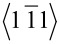 depends on step structure, which implies that 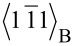 and 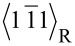 have a different CPD. To measure this difference a high measurement resolution is required.

**Figure 5:**
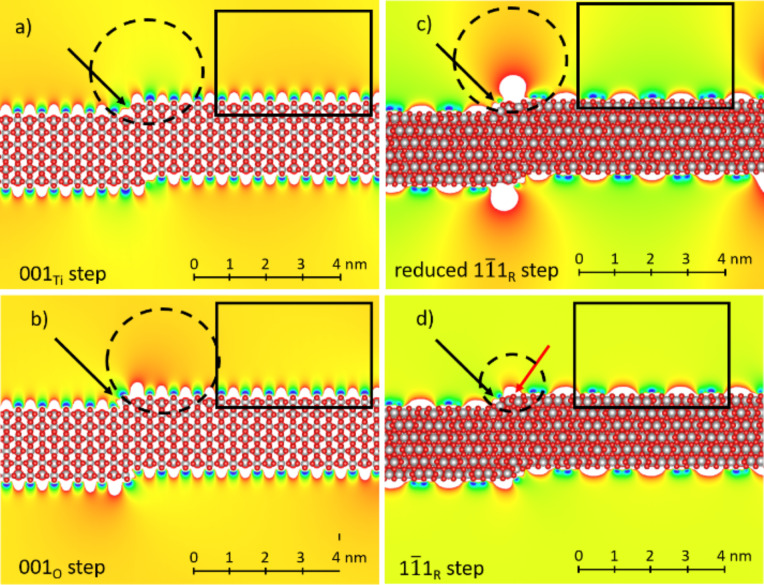
DFT simulation of the local electrostatic potential for the a) 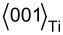, b) 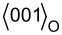, c) reduced 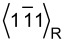, and d) 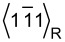 steps. Black arrows show the position of the step, the dashed ellipse and rectangle highlight the step and the (110) terrace, respectively. The red arrow in d) indicates the closest bridging oxygen atom that was removed in c) to produce the reduced 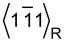 step configuration. Please note that the local potential inside the black rectangle does not have the same values because of different areas and geometries were considered for the different step configurations. The color code has the same absolute scale across all step configurations. Red indicates smaller CPD values, while blue indicates larger CPD values.

To corroborate the our assumptions, we performed DFT simulations and calculated the local electrostatic potential for four different step configurations, 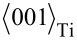, 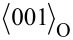, reduced 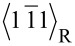, and 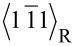, see Figure 5. Interestingly, starting from 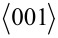, the 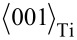 step, see Figure 5a, shows the opposite local potential and charge redistribution (dashed ellipse) compared with the (110) terrace (black rectangle). This results in a brighter contrast than in the experiment. The 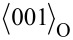 step configuration, see Figure 5b, shows a consistent charge redistribution and dipoles formation, in which the step configuration produces a pattern of positive charges pinned at five-fold coordinated Ti atoms as a result of the sideward movement of bridging oxygen atoms at the step edge. We can use those results as an indirect proof that the 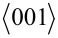 step termination is the bridge-oxygen terminated 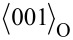. To make a comparison to the 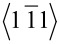 step, we begin with the 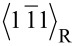 termination, see Figure 5d. Again, a positively charged area is created (small dashed ellipse) but it is much weaker than that of the 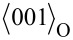 step configuration. We did not perform the calculation for the 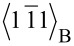 step, because it has the same in-plane oxygen termination-step geometry as 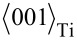. We rather examine the reduced 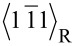 step configuration, containing an oxygen vacancy directly at step position. This configuration was observed in [35] and is also theoretically consistent [24–25,61]. The LDOS for bridging oxygen closed to the step is localized closely to the top of the valence band compared to a more distant oxygen atom, resulting in decrease of oxygen vacancy formation energy and consequent vacancy migration towards the 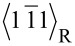 step. This geometry produces a massive positive charge pinned around a vacancy, see Figure 5c, which is higher than that at the 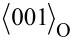 step. This is consistent with the higher drop in CPD voltage across the 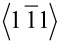 step. For simulation details see Supporting Information File 1.

## Conclusion

We performed CPD measurements around the steps on a TiO_2_(110)-(1 × 1) surface after O_2_ exposure using KPFM to investigate the local surface potential at the steps. The CPD images clearly showed that the CPD decreased at the steps, indicating that the work function locally decreased at the steps. Moreover, the reduction in work function at the 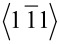 steps was larger than that at the 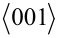 steps. We propose a plausible model for interpreting the change in work function at the steps by combining the upward dipole moment, in analogy to the Smoluchowski effect, and the local dipole moment of the surface atoms. This dipole moment is considered to lower the potential barrier for the adsorption of particular molecules, helping to suppress electron–hole recombination, increasing the quantum yield for the chemical reaction, and enhancing the catalytic reactivity. These results demonstrate novel properties of steps regarding the charge distribution, except for low coordination. The proposed dipole model should not be unique to TiO_2_ and should be valid for other catalytic materials. Therefore, this study provides insight into the important role of the steps in catalytic reactions.

## Supporting Information

File 1 Details of the DFT calculations.
