# Supplementary material for: Imaging the surface potential at the steps on the rutile TiO2(110) surface by Kelvin probe force microscopy
Source: Beilstein J Nanotechnol. 2019 Jun 13;10:1228–36. doi: 10.3762/bjnano.10.122 (PMC6604711; doi:10.3762/bjnano.10.122)
Supplement: File 1 — Details of the DFT calculations. [file Beilstein_J_Nanotechnol-10-1228-s001.pdf]

BEILSTEIN JOURNAL OF NANOTECHNOLOGY

Supporting Information

for

Imaging the surface potential at the steps on the rutile $TiO_2(110)$ surface by Kelvin probe force microscopy

Masato Miyazaki, Huan Fei Wen, Quanzhen Zhang, Yuuki Adachi, Jan Brndiar, Ivan Štich, Yan Jun Li and Yasuhiro Sugawara

Beilstein J. Nanotechnol. **2019,** *10,* 1228–1236. doi:10.3762/bjnano.10.122

Details of the DFT calculations

License and Terms: This is a supporting information file under the terms of the Creative Commons Attribution License (http://creativecommons.org/licenses/by/4.0). Please note that the reuse, redistribution and reproduction in particular requires that the authors and source are credited.
The license is subject to the *Beilstein Journal of Nanotechnology* terms and conditions: (https://www.beilstein-journals.org/bjnano)

DFT calculations were performed using VASP [1], with the standard PAW pseudopotential (4/6 electrons for Ti/O species), 400 eV plane wave cut-off, and the GGA+U [2] exchange–correlation approximation (U set to 2.5 eV). We use 1 × 6 × 1/1 × 4 × 1 k-point sampling of the Brillouin zone for the $\langle 001 \rangle / \langle 1\bar{1}1 \rangle$ step configurations. To construct the different step configurations, we follow the calculations in [3,4]. We use a much larger $\langle 1\bar{1}0 \rangle$ in-plane supercell size to converge the charge redistribution to be able to compare the $\langle 1\bar{1}0 \rangle$ area for different step configurations. Top and side views of the supercells used are shown in Figure 1. We use a six-layer configuration with opposite step configurations to neglect interactions. We use roughly 50 Å separation in the *z*-direction.

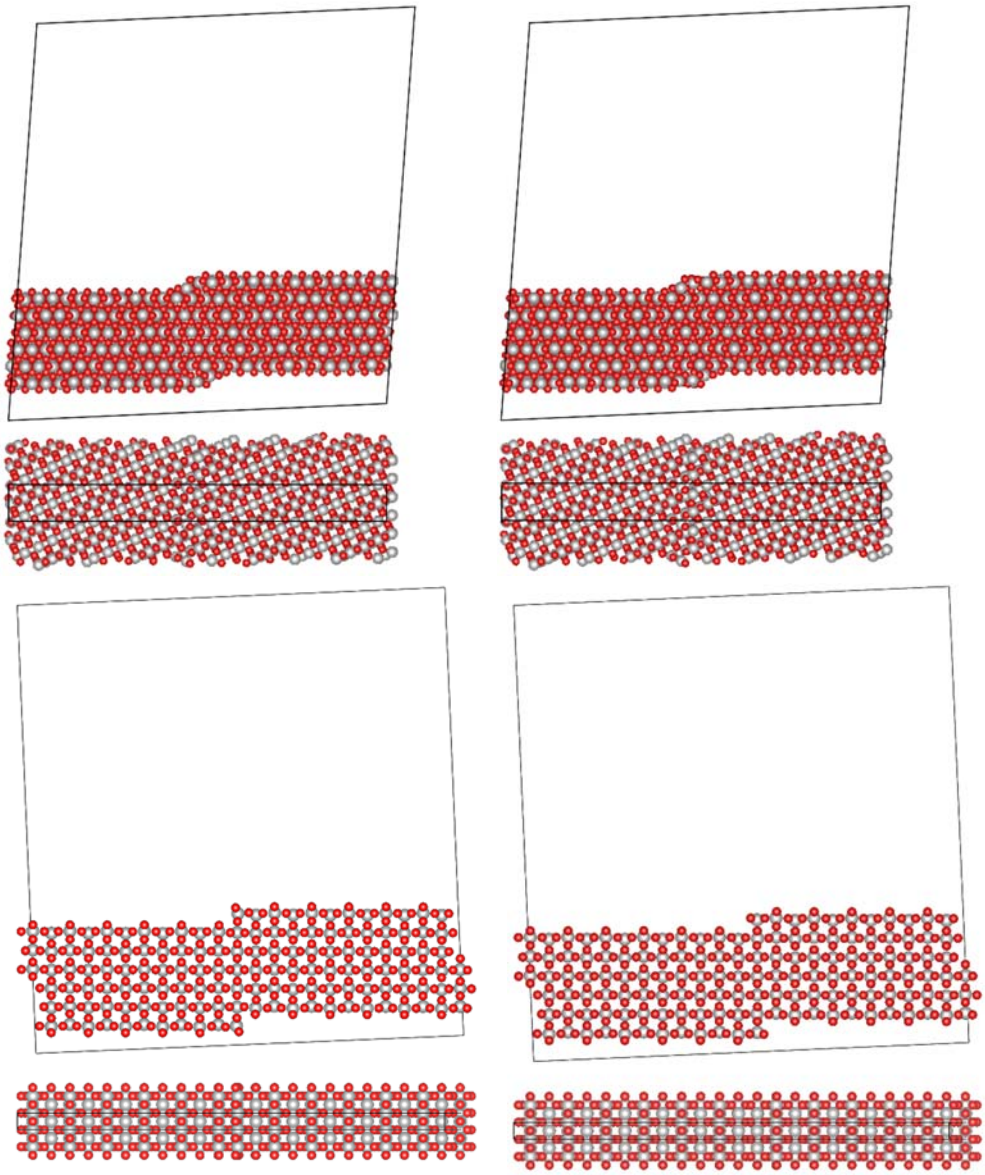

Figure 1: DFT simulation supercells for the different step configurations. From top to bottom and left to right $\langle 1\bar{1}1\rangle_R$, reduced $\langle 1\bar{1}1\rangle_R$, $\langle 001\rangle_O$, $\langle 001\rangle_{Ti}$

References

1. The VASP site. https://www.vasp.at (accessed May 16, 2019).
2. Dudarev, S. L.; Botton, G. A.; Savrasov, S. Y.; Humphreys, C. J.; Sutton, A. P. *Phys. Rev. B* **1998,** *57,* 1505. doi:10.1103/PhysRevB.57.1505
3. Stausholm-Møller, J.; Kristoffersen, H. H.; Martinez, U.; Hammer, B. *J. Chem. Phys.* **2013,** *139,* 234704. doi:10.1063/1.4840515
4. Hardcastle, T. R.; Seabourne, C. R.; Brydson, R. M. D.; Livi, K. J. T.; Scott, A. J. *J. Phys. Chem. C* **2013,** *117,* 23766. doi:10.1021/jp4078135
